# A Network of Processes for Biorefining Burdock Seeds and Roots

**DOI:** 10.3390/molecules29050937

**Published:** 2024-02-21

**Authors:** Luigi di Bitonto, Enrico Scelsi, Massimiliano Errico, Hilda Elizabeth Reynel-Ávila, Didilia Ileana Mendoza-Castillo, Adrián Bonilla-Petriciolet, Marcos Lucio Corazza, Luis Ricardo Shigueyuki Kanda, Martin Hájek, Roumiana P. Stateva, Carlo Pastore

**Affiliations:** 1 Water Research Institute (IRSA), National Research Council (CNR), Viale De Blasio 5, 70132 Bari, Italy; luigi.dibitonto@ba.irsa.cnr.it (L.d.B.); enrico.scelsi@ba.irsa.cnr.it (E.S.); 2 Department of Green Technology, Faculty of Engineering, University of Southern Denmark, Campusvej 55, 5230 Odense, Denmark; maer@igt.sdu.dk; 3 Consejo Nacional de Humanidades, Ciencias y Tecnologías (CONAHCYT), Ciudad de México 03940, Mexico; helizabeth_00@hotmail.com (H.E.R.-Á.); didi_men@hotmail.com (D.I.M.-C.); 4 Department of Chemical Engineering, Instituto Tecnológico de Aguascalientes, Aguascalientes 20256, Mexico; petriciolet@hotmail.com; 5 Department of Chemical Engineering, Universidade Federal do Paraná (UFPR), P.O. Box 19011, Curitiba 81531-980, PR, Brazil; corazza.marcos@gmail.com (M.L.C.); kanda@ufpr.br (L.R.S.K.); 6 Department of Physical Chemistry, Faculty of Chemical Technology, University of Pardubice, Studentská 95, 532 10 Pardubice, Czech Republic; martin.hajek@upce.cz; 7 Institute of Chemical Engineering, Bulgarian Academy of Sciences, Acad. G. Bonchev Str. 103, 1113 Sofia, Bulgaria; thermod@bas.bg

**Keywords:** *Arctium lappa*, burdock seeds, burdock roots, biomass valorization, enzymatic hydrolysis, ethyl levulinate, 5-hydroxymethylfurfural, circular economy

## Abstract

In this work, a novel sustainable approach was proposed for the integral valorisation of *Arctium lappa* (burdock) seeds and roots. Firstly, a preliminary recovery of bioactive compounds, including unsaturated fatty acids, was performed. Then, simple sugars (i.e., fructose and sucrose) and phenolic compounds were extracted by using compressed fluids (supercritical CO_2_ and propane). Consequently, a complete characterisation of raw biomass and extraction residues was carried out to determine the starting chemical composition in terms of residual lipids, proteins, hemicellulose, cellulose, lignin, and ash content. Subsequently, three alternative ways to utilise extraction residues were proposed and successfully tested: (i) enzymatic hydrolysis operated by Cellulases (*Thricoderma resei*) of raw and residual biomass to glucose, (ii) direct ethanolysis to produce ethyl levulinate; and (iii) pyrolysis to obtain biochar to be used as supports for the synthesis of sulfonated magnetic iron-carbon catalysts (Fe-SMCC) to be applied in the dehydration of fructose for the synthesis of 5-hydroxymethylfurfural (5-HMF). The development of these advanced approaches enabled the full utilisation of this resource through the production of fine chemicals and value-added compounds in line with the principles of the circular economy.

## 1. Introduction

*Arctium lappa*, also known as burdock, is a perennial edible plant belonging to the family *Asteraceae*. It was originally cultivated in Asia and Europe for its pharmaceutical and therapeutic properties [1,2,3] and was also used as a nutritious food. Nowadays, it has become an invasive weed on soils characterised by high nitrogen content, mainly in North American countries and Australia [4,5]. In recent years, several studies have demonstrated the beneficial effects of burdock due to the presence of various chemical compounds, including polyunsaturated fatty acids, phenolic acids, polyphenols, aldehydes, terpenoids, phytosterols, and mono- and sesquiterpenes [6,7,8]. Therefore, at present, efforts are focussed on developing methods for recovering those valuable compounds from raw biomass. In conventional extraction methods such as maceration, percolation, and Soxhlet extraction, the starting material is heated at high temperatures in the presence of organic solvents [9,10,11]. This allows higher extraction yields but, at the same time, leads to partial degradation of the thermolabile compounds, which reduces both the quality of the extract and the solid residue obtained at the end of the extraction process. In addition, they require a longer exposure time and higher energy consumption. For these reasons, novel “green technologies” have been developed that enable the extraction of bioactive compounds without the use of toxic chemicals, thus protecting the environment and human health, e.g., ultrasound, microwave-assisted, high-pressure homogenisers, enzymatic, and solid-liquid extraction using conventional and non-conventional solvents (biosolvents, supercritical fluids, and combinations of them) [12,13,14,15,16]. Extraction with compressed fluids is an example of a sustainable and efficient technique for the recovery of bioactives and secondary metabolites from raw biomass, since it prevents thermolabile compounds from degradation during the extractive step. In a recent study conducted by Stefanov et al. [13], supercritical carbon dioxide (scCO_2_) and compressed propane were successfully tested in the extraction of fatty acids and phenolic compounds from burdock seeds and roots, and the possible use of ethanol and methanol-water as cosolvents was evaluated. The extracts obtained are characterised by high antioxidant activity and could find application in the chemical and pharmaceutical fields for the formulation of nutraceuticals and cosmetics. Thus, a sustainable pathway to the valorisation of this resource is identified. However, to fully exploit its potential, extraction residues obtained at the end of the recovery process should also be used in a sustainable way [12]. The latter represents a valuable source of hemicellulose, cellulose, and lignin that could be used to produce either high-value-added compounds or as supports for the design of new bioderived catalysts to be applied in industrial processes.

In this study, for the first time, three alternative routes to convert *A. lappa* biomass have been successfully tested, namely (i) enzymatic hydrolysis to glucose, (ii) direct ethanolysis of structural carbohydrates to produce ethyl levulinate, and (iii) pyrolysis to produce biochar as a support for the synthesis of sulfonated magnetic-iron-carbon catalysts (Fe-SMCC) to be applied in the dehydration of fructose for the synthesis of 5-hydroxymethylfurfural (5-HMF).

Considering the high content of hemicellulose and cellulose in the analysed samples (about 30–50% of the total composition), *A. lappa* biomass can be used as a potential source of glucose to be applied as a substrate to produce bioethanol as well as for the synthesis of new chemical compounds [17,18,19,20,21]. Generally, this conversion is carried out by the hydrolysis of complex carbohydrates (hemicellulose and cellulose), through mechanical and/or chemical treatments with concentrated acids (HCl, H_2_SO_4_) [22,23,24], alkaline solutions [25,26] and ionic liquids [27,28]. However, these applications require drastic conditions (100–140 °C) that result in partial loss of the product and increased overall energy consumption. In this context, enzymatic hydrolysis is becoming one of the most widely used methods as it requires less energy consumption and mild environmental conditions [29,30,31]. In addition, the absence of strong acids or bases does not adversely affect the subsequent conversion processes, such as fermentation, for the formation of valuable products. For these reasons, the biomass analysed was directly tested in enzymatic hydrolysis for glucose production.

In addition, with the gradual depletion of fossil fuels, the use of structural carbohydrates in biomass for the sustainable production of biofuels and chemicals has gained increasing interest in recent years. Several high-value monomers, including 5-HMF, levulinic acid, and γ-valerolactone, can be obtained by the catalytic conversion of cellulose and hemicellulose [32,33,34]. Among these, ethyl levulinate is a versatile chemical used in industry as an eco-friendly solvent, plasticiser, perfume, liquid hydrocarbon fuel, and petroleum additive [35,36,37]. Furthermore, it is a precursor for the preparation of more complex chemicals by means of hydrolysis reactions, condensation, or other chemical reactions [38,39]. The synthesis of ethyl levulinate involves the direct acid-catalysed ethanolysis of raw biomass in the presence of homogeneous and heterogeneous catalysts. Mineral acids such as HCl and H_2_SO_4_ were widely used to produce levulinate esters in high yields [40,41]. However, these catalysts also had obvious shortcomings, such as corrosion, product separation, and catalyst recycling. Furthermore, they also promote the formation of humins as a result of secondary side reactions of hydrolysis and/or condensation [42]. Heterogeneous acid catalysts can be easily separated and reused at the end of the reaction cycle. Several catalysts, such as heteropolyacids, metal oxides, zeolites, and ionic liquids, were used to produce ethyl levulinate [43,44,45]. However, the synthesis of these catalysts turns out to be very expensive and requires long reaction times, adversely affecting the cost of the final product. In recent studies by di Bitonto et al. [46], the use of AlCl_3_·6H_2_O and H_2_SO_4_ at low concentrations (1 wt%) was found to be particularly effective in the direct conversion of municipal food waste by obtaining an ethyl levulinate yield of 60 mol% under very mild conditions (180 °C, 4 h). Experimental studies have clearly shown that the presence of H_2_SO_4_ (Brønsted acid) enables the depolymerisation of hemicellulose and cellulose into monomeric units with the formation of ethyl glucoside, while AlCl_3_·6H_2_O promotes its subsequent isomerisation into ethyl fructoside, which is easily converted to ethyl levulinate [47]. 5-Ethoxymethylfurfural (5-EMF) is a useful intermediate considered a promising alternative to conventional fossil fuels for its high stability and energy density [48,49]. The combined use of Brønsted and Lewis acids in the esterification process, therefore, not only allows for high yields but also represents a useful and economical route for the production of ethyl levulinate from lignocellulosic biomass.

Sulfonated carbon catalysts are considered promising alternatives to conventional homogeneous acid catalysts because they are inexpensive, less corrosive, and have a low environmental impact [50,51]. They can be obtained by the sulfonation of a variety of carbon-based materials, including carbon nanotubes [52], graphene [53], mesoporous carbons [54], and polyaromatic molecules [55]. To date, the use of biomass for the production of catalytic materials is becoming increasingly attractive due to its valuable advantages [56,57]. The presence of functionalised groups -SO_3_H on the surface, obtained by simple treatment with concentrated sulfuric acid, makes them particularly versatile catalysts in several industrial contexts. In addition, the porous carbon-based structure significantly increases the surface area of the catalysts, thus improving their catalytic activity. Sulfonated carbon catalysts can be easily recovered by filtration or centrifugation; however, these processes have partial separation efficiency and high energy consumption. Magnetic separation is widely used to improve catalyst recovery [58]. The introduction of magnetic materials such as Fe_3_O_4_ and Fe onto the carbon-sulfonate surfaces and the evaluation of the relevant catalytic properties in converting fructose into 5-Hydroxymethylfurural (5-HMF) were investigated. 5-HMF is a platform molecule that finds several applications in the synthesis of fine chemicals, polymers, biofuels, and pharmaceuticals [59,60]. Components of cellulose and other polysaccharides such as glucose and fructose can be converted into 5-HMF by dehydration reactions in the presence of homogeneous acid catalysts such as organic acids [61], ionic liquids [62], and mineral acids [63]. However, these processes have several drawbacks in terms of sustainability, including corrosion of the equipment, high toxicity, and recovery of the catalyst. Heterogeneous acid catalysts like supported heteropolyacids [64], zeolites [65], metal oxides [66], and ion-exchange resins [67] are gradually becoming more competitive as they are less corrosive and easily recoverable at the end of the process.

The development and consequent implementation of those routes will allow the full exploitation of this abundant resource through eco-sustainable processes with low environmental impact. Thus, affordable and truly sustainable valorisation alternatives within the concepts of the circular economy approach will be realised as a single process or in an optimised network of processes.

## 2. Results

### 2.1. Chemical Characterisation of Raw Biomass and Extraction Residues

Preliminary characterisation of raw biomass (burdock seeds and roots) and residual solids obtained after the extraction of bioactive compounds with compressed fluids was carried out to determine the starting chemical composition and identify the most valuable components to be exploited. The results are reported in Table 1.

Burdock seeds are characterised by a high lipid and protein content of 17.4 ± 0.6 and 29.7 ± 0.6 wt%, respectively. At the same time, the lipid component is almost absent in burdock roots (0.3 wt%), with proteins and lignin being the main constituents with values of 21.6 ± 0.8 and 23.1 ± 0.9 wt%, respectively.

After the extraction process, a partial enrichment of the easily hydrolysable sugars (EHS), cellulose, and lignin content in the residues produced at the end of the process was observed in all extraction residues analysed (samples 1–6). Treatment with compressed fluids to extract bioactive components such as unsaturated fatty acids and polyphenols leads to a partial deconstruction of the original structure and, thus, to a partial breaking of the intermolecular bonds that link these molecules to the raw biomass. As a result, the solids remaining at the end of the extraction process consist mainly of EHS (17.9–21.9 wt%), cellulose (19.2–26.7 wt%), and lignin (16.5–28.7 wt%). In detail, as for roots in particular, a significant content of oligo-fructose chains and inulins was determined (3–5%). These biomass residues can be used as a potential resource to be utilised further since they could easily be converted into fine chemicals and value-added compounds.

### 2.2. Study of Enzymatic Hydrolysis for Glucose Production

In this study, the commercial cellulase Novozymes Cellic^®^ CTec2 was used for its well-known ability to easily break the β 1–4 glycosidic bond of complex carbohydrates (hemicellulose, cellulose) with the formation of monomeric units [29]. The effect of enzyme loading (5 and 30 FPU/g_substrate_) and reaction time (24, 48, and 72 h) on the degradation yield was evaluated and compared with the results of enzymatic hydrolysis of commercial cellulose. The results obtained are shown in Table 2.

Using 5 FPU/g_substrate_ of the loaded enzyme, partial hydrolysis of all substrates was observed, achieving a degradation yield of about 25–40% at 50 °C after 24 h of reaction. When the reaction time was extended to 72 h, a further increase in yield was attained, reaching values of 45–70%. A comparison with experimental data on the enzymatic hydrolysis of cellulose (88.2%) reveals a partial slowdown in the catalytic activity of the enzyme used. This inactivation could be due either to the formation of enzyme-substrate complexes with other molecules present or to the adsorption of the enzyme on non-cellular components such as lignin. In any case, the increase of the loaded enzyme to 30 FPU/g_substrate_ allows for easy conversion of the hemicellulose and cellulose present in glucose by obtaining degradation yields of up to 80–100%. However, it is worth noting that burdock residues from extraction with scCO_2_ + EtOH (or MeOH in the case of roots) at 40 °C and 200 bar were not only unaffected but were even beneficial regarding hydrolysis kinetics when compared with data collected using the original biomass samples. This result is of utmost importance because the perfect synergy between an extraction with compressed fluids and the enzymatic hydrolysis of residues is demonstrated to its fullest.

### 2.3. Direct Ethanolysis for the Synthesis of Ethyl Levulinate

The residual biomass samples recovered after the corresponding extraction processes described in Table 2 were tested in direct ethanolysis for the synthesis of ethyl levulinate by using AlCl_3_·6H_2_O and H_2_SO_4_ as catalysts.

**Figure 1:**
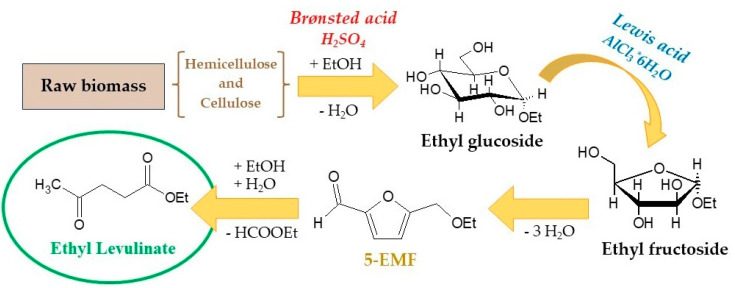
Reaction mechanism proposed for the conversion of raw biomass to ethyl levulinate by using H_2_SO_4_ and AlCl_3_·6H_2_O as catalysts [47].

**Figure 2:**
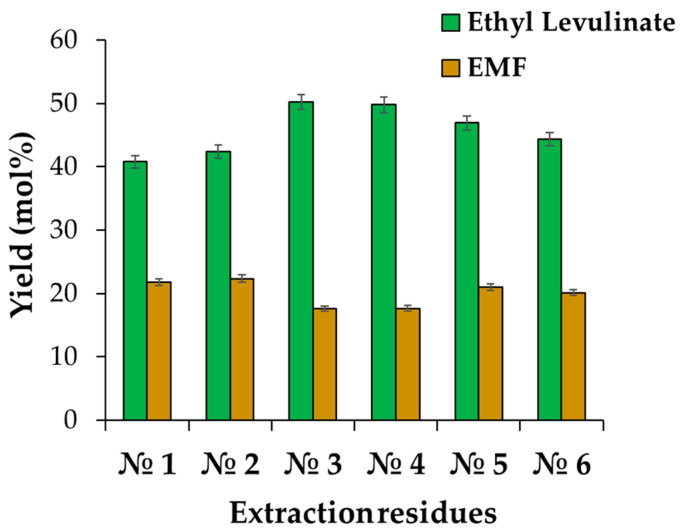
Experimental results obtained from the direct ethanolysis of the extraction residues by using AlCl_3_·6H_2_O and H_2_SO_4_ as catalysts. Reaction conditions: 0.85 g of sample, molar ratio starting carbohydrates:acid ethanolic solution: AlCl_3_·6H_2_O = 1:230:0.4, 3.5 g of CO_2_, 190 °C, 2.5 h, 300 rpm.

The mechanism and the results of the experimental tests conducted at 190 °C for 2.5 h in the presence of CO_2_ (3.5 g) are shown in Figure 1 and Figure 2, respectively.

From the treatment of residual solids, a yield of 40–50%mol of ethyl levulinate was obtained with a content of 5-EMF of 18–20%mol. As a result, about 60–70% of carbohydrates present in the starting biomass are converted into useful products. Consequently, the application of this process for the utilisation of residual biomass thus represents a rapid advance towards the development of an eco-sustainable economy in which the extraction residues resulting from the extraction of bioactive compounds operated through supercritical fluids can be converted into biofuels and green solvents to be applied in different industrial contexts.

### 2.4. Synthesis and Characterisation of Iron Sulfonated Magnetic Carbon Catalysts

**Figure 3:**
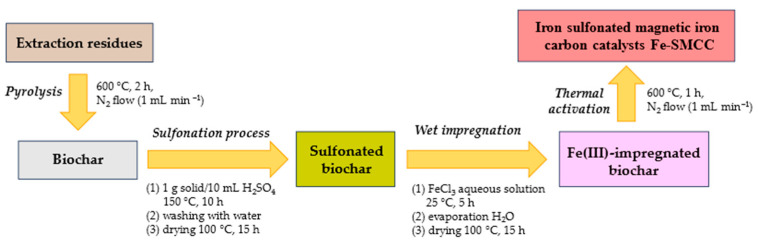
Synthetic route for the synthesis of iron sulfonated magnetic carbon catalysts (Fe-SMCC) from the extraction residues of Burdock seeds and roots.

The extraction residues recovered from *A. lappa* seeds and roots were used for the synthesis of iron sulfonated magnetic carbon catalysts (Fe-SMCC). A schematic illustration of the synthetic route for Fe-SMCC is shown in Figure 3.

**Figure 4:**
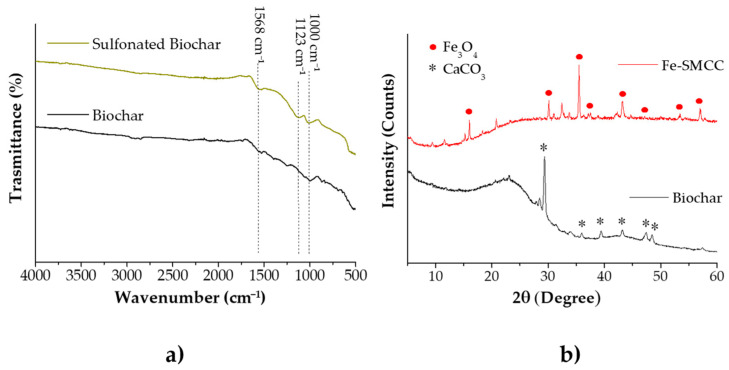
(**a**) Comparison of FTIR spectra of biochar and biochar obtained after the sulfonation process. (**b**) XRD spectra of biochar and iron-supported magnetic carbon catalyst (Fe-SMCC).

The carbonaceous material (biochar) was obtained from the pyrolysis of the extraction residues at high temperatures (600 °C, 2 h) under nitrogen (N_2_) flow. Subsequently, the biochar obtained was treated with concentrated sulfuric acid (150 °C, 10 h), resulting in the formation of strongly acidic sulfonic groups on the catalyst surface. Figure 4a shows the FTIR spectra of biochar and sulfonated biochar. Both samples exhibit a typical signal at 1568 cm^−1^, attributable to the stretching signal of C=C bonds of the aromatic rings present in the carbonised materials deriving from lignocellulosic biomass. After the sulfonation process, the presence of new signals at 1123 and 1100 cm^−1^ evidences the incorporation efficiency of -SO_3_H groups on the biochar surface [68,69].

**Figure 5:**
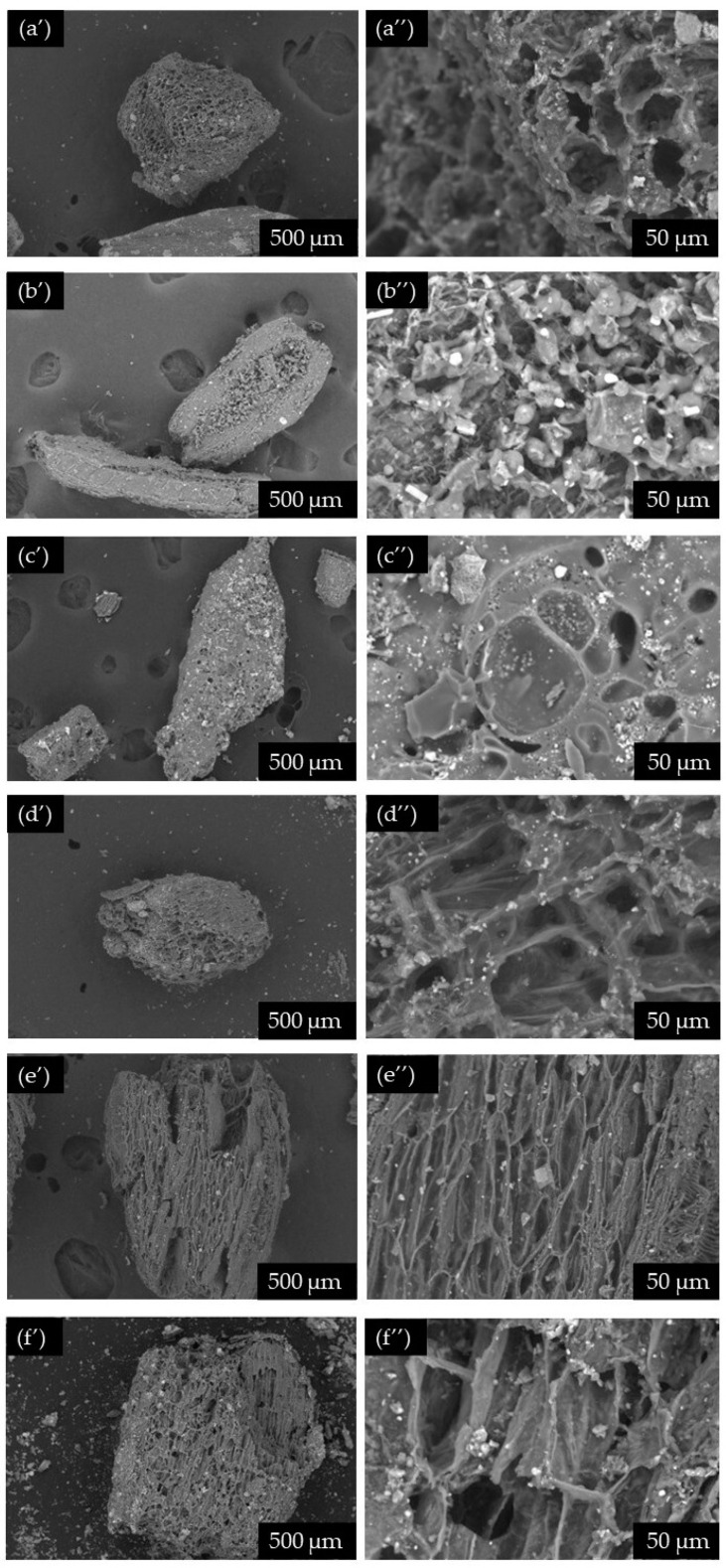
SEM images of biochar ((**a’**,**a’’**) of seeds and (**d’**,**d’’**) of roots, respectively), sulfonated biochar ((**b’**,**b’’**) for seeds and (**e’**,**e’’**) for roots), and Fe-SMCC ((**c’**,**c’’**) for seeds and (**f’**,**f’’**) for roots).

Then, the sulfonated biochar was impregnated with an aqueous solution of iron (III) chloride (25 °C, 5 h) and converted into Fe-SMCC by further thermal activation (600 °C, 1 h, N_2_). Figure 4b reports the XRD patterns of the starting biochar and Fe-SMCC. The presence of a weak broad diffraction peak at 2θ of ~23–28° in both spectra can be attributed to amorphous carbon structures containing aromatic carbon sheets [70]. In addition, signals located at 2θ of 29.3°, 35.9°, 43.0°, 47.4°, and 48.5° have identified the presence of calcium carbonate (CaCO_3_) in the starting biochar. The diffraction peaks in Fe-SMCC at 2θ of 16.1°, 30.2°, 35.5°, 37.7°, 43.2°, 47.5°, 53.5°, and 57.1° have highlighted the formation of magnetite Fe_3_O_4_ on the catalyst, confirming the effectiveness of the synthesis process. The SEM images of biochar, biochar sulfonate, and Fe-SMCC are shown in Figure 5.

Biochar (Figure 5a) exhibits an irregular and heterogeneous morphology with a well-developed alveolar structure resulting from the loss of volatile compounds during heat treatment [71]. After the sulfonation process (Figure 5b), a partial reduction in porosity was observed on the surface of the carbonaceous material. This effect is related to the partial removal of some parts of the amorphous carbon present in the starting biochar after the chemical treatment with sulfuric acid [72,73]. At the same time, the binding of -SO_3_H groups on the surface of biochar involves a partial reduction in the porosity of the support. Nanoparticles of Fe_3_O_4_ were observed on the surface of Fe-SMCC at the end of the process (Figure 5c’ ), obtained as a result of wet impregnation of sulfonated biochar with FeCl_3_ and the subsequent activation process. The effectiveness of the synthesis process was also confirmed by elementary analyses. Table 3 shows the elementary compositions obtained by EDS analysis of biochar, biochar sulfonate, and Fe-SMCC for both types of extraction residues (burdock seeds and roots).

The chemical composition of biochar is clearly different before and after the sulfonation process. The increase in sulphur (S) and oxygen (O) content by 2.34–2.64 wt% and 19.7–26.8 wt%, respectively, indicates the correct incorporation of sulfonic groups on the biochar surface. The presence of iron (Fe) in sulfonated materials after the impregnation, washing, and thermal activation of 4.18–5.51 wt%, confirmed the formation of magnetite in Fe-SMCC. Finally, the total amount of active sites present on the surface of the synthesised catalysts was determined by the Boehm titration method [74] (Table 4), resulting in an overall acid density between 0.74 and 1.36 mmol_SO_3_H_/g.

#### Dehydratation of Fructose for the Synthesis of 5-HMF Catalysed by Fe-SMCC

**Figure 6:**
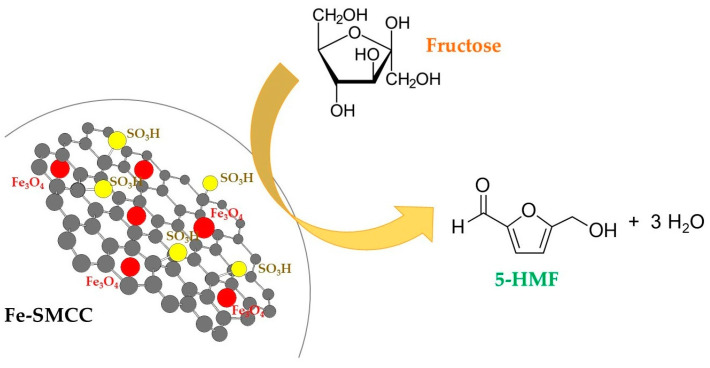
Use of Fe-SMCC in the dehydration of fructose for the synthesis of 5-HMF.

Fe-SMCC was tested in the dehydration of fructose for the synthesis of 5-HMF (Figure 6).

Although water is the most environmentally friendly solvent, fructose dehydration in an aqueous medium is generally not selective, generating hydrolysis and/or condensation by-products (i.e., humins) [59,60]. In order to improve the yield of 5-HMF, the dehydration reaction was carried out by using methyl isobutyl ketone (MIBK) and γ-valerolactone (GVL) as cosolvents. In addition, the catalytic activity of commercial resin (Amberlyst-15) in the dehydration process was also investigated. The results obtained are reported in Table 5.

**Figure 7:**
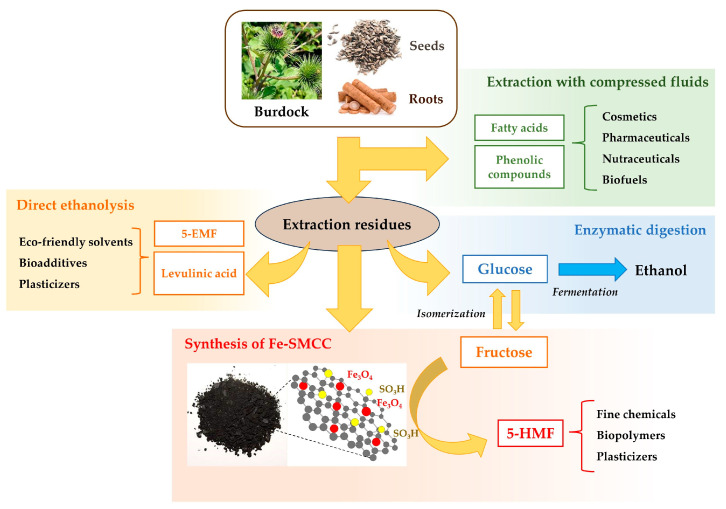
Integrated biorefinery approach for the complete valorisation of burdock seeds and roots.

Under the reaction conditions adopted (0.2 mmol fructose, organic phase = MIBK, volume ratio MIBK to H_2_O = 2 to 1, 130 °C, 6 h), in the absence of a catalyst, a fructose conversion of 28.8 ± 1.2% was obtained with a 5-HMF yield of 6.6 ± 0.3% and a selectivity of 22.9 ± 2.0% (Entry 1). When Amberlyst-15 was used as a catalyst, the fructose conversion was 91.8 ± 2.3% with a 5-HMF yield of 14.5 ± 0.5%mol. However, the selectivity towards the production of 5-HMF was found to be only 15.8 ± 0.8 mol%, lower than the non-catalysed process. The acidic active sites present on the catalyst surface [67] induced further hydrolysis of the 5-HMF by generating formic and levulinic acid as reaction by-products (32.3 ± 1.6 and 28.6 ± 1.4 mol%, respectively). On the other hand, the results obtained using Fe-SCMM (Entries 3–8) showed that, despite the lower conversion of fructose of 52.5–62.5 mol%, the systems tested were more active and selective towards the production of 5-HMF, with values in the range of 16.2–23.1 mol% and 30.1–42.0 mol%, respectively, confirming the fundamental role of the catalysts in the selective dehydration process. Compared to Amberlyst-15, which contains only sulfonic groups (4.3 mmol_SO_3_H_/g, as experimentally determined), the simultaneous presence of Brønsted (sulfonic groups) and Lewis (magnetite) acid sites allows to effectively catalyse the dehydration reaction, obtaining 5-HMF as the main product. These values can be further improved by using GVL as a cosolvent reaction (entries 9–14), with yields in 5-HMF of 28.9–41.9 mol% and selectivity of 40.1–55.5 mol%. However, in the case of MIBK, a convenient separation of 5-HMF was obtained at the end of the dehydration process (Figure 7). More than 70% of 5-HMF was present in the higher organic phase (R = 2.4–2.9), allowing easy recovery and isolation by distillation [66].

## 3. Discussion

### Biorefinery Approach for the Integral Valorisation of Burdock Seeds and Roots

*A. lappa* seeds and roots have always attracted attention for their therapeutic and pharmaceutical properties. Over the years, as an alternative to their direct consumption, several studies have mainly focused on the extraction of bioactive components (e.g., fatty acids, polyphenolic compounds) to be used in the manufacture of various products, such as nutraceuticals and pharmaceuticals. However, there is still a gap regarding the fate of extraction residues, which are supposed to be disposed of, since till now, it has not been demonstrated how they can be utilized on an industrial scale. Inevitably, that would lead to an increase in economic costs and a significant impact on the environment. In detail, considering that extractable components represent only about 15–20% of the total biomass, the possible conversion of the residual biomass to value added compounds will have a crucial impact on the overall feasibility evaluation. Moreover, hemicellulose, cellulose, inulin-type fructans in the case of roots [4,75] and lignin, which are the main constituents, can be exploited for the production of fine chemicals to be used in several other fields, namely new materials and liquid biofuel. The development of an integrated biorefinery approach for the full exploitation of burdock biomasses would not only reduce the costs of disposal but convert them from an environmental burden to a valuable resource, in accordance with the principles of the circular economy. The conceptual process diagram suggested for the valorisation of burdock seeds and roots is shown in Figure 7. As shown, the first step in an integrated biorefinery is the recovery of bioactive components using compressed liquids (supercritical CO_2_ and propane). The extraction residues obtained are mainly composed of hemicellulose (17.9–21.9 wt%), cellulose (19.2–26.7 wt%), inulin-type fructans in the case of roots (2–5%), and lignin (16.5–28.7 wt%), as reported in Table 1.

Several sustainable methods are studied to enable the full exploitation of these residual biomasses in a multi-product biorefinery scheme, in which bio-ethanol, 5-HMF, 5-EMF, levulinic acid, and ethyl levulinate could eventually be obtained.

The first process investigated was the enzymatic digestion of the extraction residues, which allowed a complete conversion of complex carbohydrates (hemicellulose and cellulose, representing about 30–50% of the total biomass) into simple sugars by obtaining a glucose yield of 90–100%. In detail, the extraction process operated through compressed fluids can even be seen as an efficient pre-treatment of biomasses since the hydrolysis’ kinetics improved by 15–25% with respect to the direct hydrolysis operated on the original burdock biomasses. The obtainment of glucose (and fructose from roots) can be functional to ethanol production through fermentation, according to well-known routes [76]. Ethanol can either be considered a final product (biofuel) or used in other processes involved in biorefining burdock residues. The best way to extract from burdock roots oligofructose species, known as prebiotic fibres, uses ethanol as a cosolvent [77]. Also, ethanol could be used in the direct alcoholysis of *A. lappa* seeds and root residues to obtain new platform molecules. In fact, operating at relatively mild conditions, namely 190 °C and 2.5 h, in the presence of very cheap catalysts, namely AlCl_3_·6H_2_O and H_2_SO_4_, about 70–80 mol% of the starting polymeric carbohydrates can be successfully converted into ethyl levulinate and 5-EMF, which will find application as bio-additives in bioderived materials. On the other hand, as a result of the enzymatic digestion of burdock seeds and roots, the glucose (and fructose in the specific case of roots) aqueous solutions could be directly used for the production of levulinic acid using an appropriate combination of catalysts (AlCl_3_·6H_2_O and H_2_SO_4_) [78], ethyl levulinate [79], or 5-HMF using the more selective heterogeneous ferromagnetic catalyst obtained from burdock seeds and root residues.

All of these options may efficiently compete and complete the flexible valorisation of burdock seeds and roots by offering a number of alternative products that can be appropriately modulated and selected following specific market requirements. With the simple inclusion of cheap and largely available reagents, namely *Cellulases*, AlCl_3_·6H_2_O, and H_2_SO_4_ and well-known processes (fermentation of sugars, alcoholysis, and thermo-chemical valorisation), an *A. lappa* biorefinery burdock could represent an opportunity to improve the present economic scenario, especially in regions and countries where the plant is already cultivated widely. For example, in Fengxian’s district, known as the “Hometown of Burdock” in China, the burdock cultivation area is over 3000 hm^2^, producing 150,000 t of roots annually [8].

Thus, the novel ideas advocated in this work about the sustainable industrial application of the processes investigated and proposed could allow a potential production of 1000–2000 t extractives, 12,000–13,000 t ethanol, 10,000 t levulinate (as acid and/or ethyl esters), and 5000 t hydroxymethylfurfural, turning a “traditional medicinal plant” into a modern competitive provider of new pharmaceuticals and superfoods, as well as fine chemicals for the production of new bioderived materials and biofuels.

## 4. Materials and Methods

### 4.1. Reagents and Instruments

All reagents were of analytical grade (≥99%) and were used directly without further purification or treatment. Fourier-transform infrared spectroscopy (FTIR) spectra were recorded on a Nicolet Summit Thermo Fisher Scientific FTIR spectrophotometer (Thermo Fisher Scientific, Waltham, MA, USA) equipped with an Everest Diamond ATR module. Scanning electron microscopy (SEM) and Energy Dispersive X-ray (EDX) spectra were performed by using a tabletop microscope, the Hitachi TM4000Plus (Hitachi, Tokyo, Japan). Carbohydrates, organic acids, and 5-HMF were determined by high-performance liquid chromatography (HPLC) with a JASCO (Easton, MD, USA) instrument equipped with a Hi-Plex H column (300 mm, 4 mm; Agilent, Santa Clara, CA, USA) and two different types of detectors: the RI-150 refractive index detector and the UV-150 detector (detection wavelengths of 235 and 260 nm). The samples were injected automatically (100 µL) with an autosampler AS 2055. The column was thermostatically controlled at 55 °C, and a 0.6 mL/min flow rate was applied, using a 0.01 M sulfuric acid (H_2_SO_4_) solution as the mobile phase. Gas-chromatographic (GC) analysis for the determination of ethyl levulinate were done with a Shimadzu GC 2010 Plus gas chromatograph equipped with an Agilent SH-Rtx-Wax capillary column (Shimadzu (Kyoto, Japan), 30 m × 0.32 mm × 0.25 μm) and a flame ionisation detector (FID). The injector and detector temperatures were set at 250 and 280 °C, respectively. The oven temperature was programmed with the following temperature ramp: 30 °C for 3 min, then increased to 240 °C at 10 °C min^−1^ and held at 240 °C for 15 min. A split ratio of 1:10 was used with Helium (He) as carrier gas (flow rate 1.0 mL/min). X-ray diffraction (XRD) analysis was carried out by using an Empyrean (Malvern169 Panalytical, Chipping Norton, Australia) diffractometer equipped with a PIXcel1D-Medipix3 detector operating with CuKα radiation (λ = 1.5406 Å, 45 kV, 40 mA) and a 2θ scanning range of 5 to 60°. The results obtained were analysed with HighScore Plus 4.8 software and the PDF2 database.

### 4.2. Biomass Characterisation

Burdock seeds were provided by a local farm in the town of Ivaipora (Parana, Brazil), while the roots were provided by a certified herbal pharmacy in Sofia (Bulgaria). Both biomasses were initially air-dried in an oven at 60 °C for 48 h, ground in a mixer (Philips Walita RI1774 (Philips, Amsterdam, The Netherlands)), and sieved to obtain a final particle size of 0.42–0.71 mm. The extraction residues (seeds and roots) were recovered after the preliminary extraction of bioactive components with compressed fluids (scCO_2_ and propane), according to the experimental procedures described by Stefanov et al. [13]. Finally, all samples were analysed in terms of residual lipids, proteins, hemicellulose, lignin, and ash content, as described by di Bitonto et al. [47]. The average chemical composition of starting biomass and extraction residues is reported in Table 1. Each analysis was carried out in triplicate, allowing the average value and the relative standard deviation to be determined.

### 4.3. Enzymatic Tests

Enzymatic hydrolysis of raw biomass and extraction residues was carried out at 50 °C under the following conditions: 250 μL of commercial cellulase (Cellic^®^ CTec2, >1000 Biomass Hydrolysis Units, density of 1.209 g/L; Novozymes, Lyngby, Denmark) were initially dissolved in citrate buffer solution (50 mmol, pH 4.8, up to 5 mL) and left for 1 h at 50 °C to activate the enzyme. Part of this solution was opportunely up-taken and diluted up to 5 mL of citrate buffer before being added to a mixture containing 0.1 g of solid substrate and 5 mL of citrate buffer to achieve the appropriate enzymatic load (5 and 30 FPU/g cellulose). The filter paper activity of the enzyme was previously experimentally determined [80]. Then, 200 µL of sodium azide solution (20 mg/mL) were added as an antimicrobial agent. The reaction was carried out at 50 °C for 72 h under constant stirring speed (300 rpm). To study the enzymatic kinetics, samples (1 mL) were withdrawn at 24, 48, and 72 h, filtered using 0.2 µm nylon filters, and analysed for the determination of hydrolysed glucose by HPLC analysis. The reaction yield was calculated according to Equation (1):

(1) $$Enzymatic\;degradation\;yield\left(\mathrm{wt}\%\right)=\frac{m_{\mathrm{hydrolysed}\;\mathrm{glucose}}}{m_{\mathrm{biomass}}{\%}_{\mathrm{cellulose}}}\frac{m_{\mathrm{hydrolysed}\;\mathrm{glucose}}}{m_{\mathrm{biomass}}{\%}_{\mathrm{cellulose}}}\;\times \;100$$

where m_hydrolyseds glucose_ is the mass of glucose experimentally detected after the enzymatic hydrolysis, m_biomass_ is the weighted amount of sample used, and %_cellulose_ is the cellulose content determined in the starting samples as reported in Table 1.

### 4.4. Ethanolysis Reaction

The direct ethanolysis of extraction residues was performed in a 50 mL stainless stirrer reactor (Parr series 4590, model 4597 (Parr Instrument Company, Moline, IL, USA)), equipped with a stirring speed controller, temperature control, and pressure indicator. In a typical reaction, 0.6 g of sample (total carbohydrate content = 43.8 wt%) were mixed with 20 mL of an ethanolic solution of 1 wt% H_2_SO_4_ and 0.14 g of AlCl_3_·6H_2_O, resulting in a final molar ratio of carbohydrate:acidic ethanolic solution:catalyst of 1:230:0.4 [46,47]. Then, the reactor was loaded with 3.5 g of CO_2_ (≥99%, Air Liquid) by using a syringe pump (Teledyne Isco model 260D (Teledyne ISCO, Lincoln, NE, USA)) held at a constant temperature (15 °C) and pressure (150 bar). The reaction was carried out at 190 °C for 2.5 h with stirring at 300 rpm. At the end of the process, the system was cooled to room temperature and depressurised by opening a needle valve (pressure release rate of about 5 bar/min). The reaction mixture was collected, and the organic solution was separated from the residual solid by centrifugation (3000 rpm, 10 min; Rotofix 32 Hettich centrifuge (Hettich Lab, Tuttlingen, Germany)), and 5-EMF content was determined spectroscopically by using a UV–Vis SP 8001 spectrophotometer (Metertech, Taipei, Taiwan) [47]. Finally, ethyl levulinate was quantified by gas chromatographic analysis of the extract (1 µL) obtained after five successive extractions of the organic mixture with heptane (5 mL). Determinations were performed by external calibration with ethyl levulinate standard solutions in heptane ranging from 10 to 1000 ppm.

### 4.5. Synthesis of Iron Sulfonated Magnetic Carbon Catalysts

Iron sulfonated magnetic carbon catalysts (Fe-SMCC) were synthesised according to the synthetic route shown in Figure 5. Firstly, biochar was obtained by pyrolysis of extraction residues at 600 °C for 2 h with a heating rate of 10 °C/min and an N_2_ flow of 1 mL/min. Subsequently, the porous carbon material was sulfonated with concentrated sulfuric acid at 150 °C for 10 h (1 g solid/10 mL H_2_SO_4_) to generate strongly acidic sulfone groups on the biochar surface. After the process completion, the sulfonated biochar was washed with deionised water until no more sulphate ions were detected in the wash water, and it was dried in an oven at 100 °C for 15 h. This activated carbon was used as a support material for the synthesis of Fe-SMCC by wet impregnation. 1 g of the sample was suspended into 50 mL of an aqueous solution of iron (III) chloride hexahydrate (1.2 g of FeCl_3_·6H_2_O, weight ratio Fe to biochar of 25%) at room temperature for 5 h. The suspension was then evaporated at 110 °C and the resultant solid dried at 100 °C for 15 h. Fe-SMCC were obtained after the thermal activation of Fe (III)-impregnated biochar at 600 °C for 1 h under N_2_ flow (1 mL/min). The Boehm titration method was used for the determination of acid properties of the catalysts synthetised [65].

#### Dehydratation Process

The synthesised catalysts were tested in the reaction of dehydration of fructose for the synthesis of 5-HMF. In a Pyrex glass reactor of 10 mL, 0.1 g of supported catalyst was mixed with 1 mL of aqueous fructose solution (0.2 mmol) and 2 mL of organic solvent (methyl isobutyl ketone and γ-valerolactone), by obtaining a final volume ratio of organic to aqueous phase of 2 to 1. The reaction was carried out at 130 °C for 6 h with stirring at 300 rpm. At the end of the process, the system was cooled to room temperature in an ice-water bath, and the resultant organic mixture was analysed by HPLC for the determination of residual fructose (RI detection), organic acids (UV detection 235 nm) and 5-HMF (UV detection 260 nm). The conversion of fructose, the yield of the reaction products and the selectivity towards the production of 5-HMF were calculated by using Equations (2)–(4):

(2) $$\mathrm{Fructose}\;\mathrm{conversion}\;\left(\mathrm{mol}\%\right)=\frac{{\mathrm{mmol}}_{\mathrm{starting}\;\mathrm{fructose}}-{\mathrm{mmol}}_{\mathrm{residual}\;\mathrm{fructose}}}{{\mathrm{mmol}}_{\mathrm{starting}\;\mathrm{fructose}}}\;\times \;100\;$$

(3) $$\mathrm{Yield}\;\mathrm{product}\left(\mathrm{mol}\%\right)=\frac{{\mathrm{mmol}}_{\mathrm{product}}}{{\mathrm{mmol}}_{\mathrm{starting}\;\mathrm{fructose}}}\times \;100\;$$

(4) $$\mathrm{Selectivity}\;5\text{-}\mathrm{HMF}\left(\mathrm{mol}\%\right)=\frac{{\mathrm{mmol}}_{5\text{-}\mathrm{HMF}}}{{\mathrm{mmol}}_{\mathrm{starting}\;\mathrm{fructose}}}\times \;100\;$$

In addition, in the case of MIBK, the partition coefficient (R) of 5-HMF between the two phases (aqueous and organic) obtained at the end of the dehydration process was determined according to Equation (5).

(5) $$R=\frac{{\mathrm{mmol}}_{5\text{-}\mathrm{HMF}\;\mathrm{organic}\;\mathrm{phase}}}{{\mathrm{mmol}}_{5\text{-}\mathrm{HMF}\;\mathrm{aqueous}\;\mathrm{phase}}}$$

## 5. Conclusions

An integrated strategy for biorefining of lignocellulosic biomasses of *Arctium lappa* burdock seeds and roots was developed and presented. A cascade of technologies was experimentally studied using as possible feedstocks the residual biomasses obtained after recovery of fatty acids and antioxidants applying compressed fluids. Valorisation routes of the residual cakes to attain glucose, ethyl levulinate and new magnetic catalysts were specifically investigated. It was demonstrated that cellulose can be almost totally hydrolysed in less than 48 h at 50 °C, by commercial enzymes into simple sugars (e.g., glucose and xylose) using a load of enzyme of 30 FPU/g_substrate_ on the residues obtained from extractions with sc-CO_2_/alcohols. Alternatively, lignocellulosic residues can be directly reacted through ethanolysis operated at 190 °C for 2.5 h in the presence of AlCl_3_·6H_2_O and H_2_SO_4_, achieving high yields of ethyl levulinate (40–50%) and 5-EMF (18–22%). Finally, novel magnetic Iron (III) based sulfonated lignin-based biochars were also synthesized, characterised and found to efficiently catalyse the fructose conversion into 5-HMF in biphasic water-MIBK or GVL systems up to obtain reasonably yields (28.9–41.9 mol%) and selectivity (40.1–55.5 mol%). All these fundamental steps can concur in defining a network of processes for a multiproduct biorefinery of *Arctium lappa* seeds and roots to obtain platform chemicals and biofuels.

## Figures and Tables

**Table 1:** Chemical characterisation of raw biomass (burdock seeds and roots) and extraction residues obtained after the preliminary extraction of bioactive compounds by using compressed fluids (scCO_2_ and propane).

Samples	Seeds					Roots		
	Raw Biomass	Extraction Residues				Raw Biomass	Extraction Residues	
		№ 1	№ 2	№ 3	№ 4		№ 5	№ 6
Solvent	-	scCO_2_ + EtOH	Propane	Propane	Propane	-	scCO_2_ + MeOH/H_2_O	scCO_2_ + MeOH/H_2_O
Extraction Conditions	-	40 °C 200 bar	40 °C 60 bar	80 °C 200 bar	Mix of T and P	-	40 °C, 200 bar	80 °C, 200 bar
Ref. in Article *J CO_2_ Util.* [13]	-	Run 2	Run 4	Run 8	-	-	Runs 15–17	Runs 18–20
Moisture content, %	10.2 ± 0.4	4.6 ± 0.2	4.5 ± 0.2	3.4 ± 0.1	3.9 ± 0.1	9.7 ± 0.3	6.2 ± 0.1	6.0 ± 0.1
Total Solids composition (wt%)								
Total Lipids	17.4 ± 0.6	6.7 ± 0.3	8.3 ± 0.3	6.2 ± 0.2	9.5 ± 0.4	0.3	-	-
Proteins	29.7 ± 1.1	17 ± 0.6	18.6 ± 0.7	14.7 ± 0.5	12.7 ± 0.5	21.6 ± 0.8	15.6 ± 0.6	14.4 ± 0.4
EHS *	18.4 ± 0.8	19.1 ± 0.7	21 ± 0.8	20.4 ± 0.7	21.9 ± 0.8	18.1 ± 0.6	17.9 ± 0.6	18.8 ± 0.7
Cellulose	13.5 ± 0.6	24.7 ± 1.0	26.7 ± 1.1	26.5 ± 0.6	24.9 ± 0.8	14.1 ± 0.6	19.2 ± 0.7	21.9 ± 1.0
Lignin	11.6 ± 0.5	19.5 ± 0.8	16.5 ± 0.6	21.2 ± 0.6	19.6 ± 0.8	23.1 ± 0.9	28.7 ± 1.2	26.5 ± 1.1
Ashes	4.7 ± 0.1	5 ± 0.1	4.9 ± 0.1	4.3 ± 0.1	4.2 ± 0.1	16.5 ± 0.6	12.6 ± 0.4	13.1 ± 0.4

* EHS: easily hydrolysable sugars (inuline, oligofructose, and hemicellulose).

**Table 2:** Enzymatic hydrolysis of raw biomass and extraction residues for glucose production. Reaction conditions: 1 g of sample, 10 mL of citrate buffer (50 mmol, pH 4.8), concentration enzyme (Novozymes Cellic^®^ CTec2), 5–30 FPU/g_substrate_, 50 °C, 72 h, 300 rpm.

Substrate	Total Carbohydrate Content (wt%)	Enzymatic Degradation Yield (wt%)						
		Time (h)	24		48		72	
		FPU/g_substrate_	5	30	5	30	5	30
Cellulose	100		71.6 ± 2.8	84.5 ± 0.9	80.8 ± 0.8	100	88.2 ± 1.9	100
Burdock seeds	31.9 ± 1.4		40.1 ± 1.3	88.2 ± 2.2	46.0 ± 1.8	93.8 ± 1.7	49.6 ± 1.0	100
№ 1	43.8 ± 1.7		43.5 ± 0.9	81.2 ± 1.6	45.2 ± 1.6	94.1 ± 1.4	46.5 ± 1.4	96.7 ± 1.8
№ 2	47.7 ± 1.9		40.9 ± 1.1	81.7 ± 1.4	44.0 ± 1.1	83.9 ± 1.5	47.1 ± 1.3	89.3 ± 1.2
№ 3	46.9 ± 1.3		25.7 ± 0.7	48.4 ± 0.6	28.3 ± 0.8	51.7 ± 0.8	29.5 ± 0.7	53.7 ± 0.8
№ 4	46.8 ± 1.6		28.5 ± 0.9	51.9 ± 0.8	27.8 ± 0.9	54.8 ± 0.7	30.2 ± 0.8	56.0 ± 1.4
Burdock roots	32.2 ± 1.2		58.5 ± 1.4	88.3 ± 1.2	68.4 ± 1.4	95.6 ± 2.1	68.5 ± 1.9	100
№ 5	37.1 ± 1.3		73.2 ± 1.8	82.3 ± 1.4	87.7 ± 1.6	87.3 ± 0.9	94.5 ± 2.1	100
№ 6	40.7 ± 1.7		75.5 ± 1.6	98.4 ± 2.5	79.5 ± 1.1	100	86.6 ± 1.6	100

**Table 3:** Elemental analysis of biochar, sulfonated biochar, and Fe-SMCC obtained from the extraction residues of Burdock seeds and roots.

Samples	Extraction Residues									
	Seeds					Roots				
Elemental Composition (wt%)	C	O	Ca	S	Fe	C	O	Ca	S	Fe
Biochar	83.0	14.2	0.96	-	-	69.3	24.8	1.6	0.32	-
Sulfonated biochar	76.8	19.7	-	2.34	-	68.2	26.8	-	2.64	-
Fe-SMCC	65.6	21.4	-	1.27	5.51	70.3	16.3	0.86	1.03	4.18

**Table 4:** Determination of acid properties for Fe-SMCC.

Catalysts	Total Acid Density (mmol_SO_3_H_/g)
Fe-SMCC № 1	0.86 ± 0.02
Fe-SMCC № 2	0.98 ± 0.03
Fe-SMCC № 3	0.95 ± 0.02
Fe-SMCC № 4	1.36 ± 0.04
Fe-SMCC № 5	1.32 ± 0.04
Fe-SMCC № 6	0.78 ± 0.02

**Table 5:** Reactivity tests for the dehydration of fructose for the synthesis of 5-HMF. Reaction conditions: 1 mL of aqueous solution of fructose (0.2 mmol), 2 mL of organic solvent (MIBK or GVL), 130 °C, 6 h, 300 rpm.

E	Solvent	Catalyst	Fructose Conversion (mol%)	5-HMF			Formic Acid	Levulinic Acid
				Yield (mol%)	Selectivity (%)	R	Yield (mol%)	Yield (mol%)
1	MIBK	In the absence of a catalyst	28.8 ± 1.2	6.6 ± 0.3	22.9 ± 2.0	2.2	-	-
2		Amberlyst-15	91.8 ± 2.3	14.5 ± 0.5	15.8 ± 0.8	2.4	32.3 ± 1.6	28.6 ± 1.4
3		Fe-SMCC № 1	53.6 ± 1.7	18.7 ± 0.3	34.9 ± 1.7	2.4	-	-
4		Fe-SMCC № 2	57.0 ± 0.5	21.1 ± 0.8	37.0 ± 1.7	2.8	-	-
5		Fe-SMCC № 3	55.0 ± 0.7	23.1 ± 0.8	42.0 ± 2.0	2.8	-	-
6		Fe-SMCC № 4	58.3 ± 0.5	21.9 ± 1.6	37.6 ± 3.1	2.9	-	-
7		Fe-SMCC № 5	52.5 ± 1.9	16.2 ± 0.6	30.9 ± 2.3	2.6	-	-
8		Fe-SMCC № 6	62.5 ± 1.4	18.8 ± 0.4	30.1 ± 1.3	2.5	-	-
9	GVL	Fe-SMCC № 1	69.0 ± 1.4	28.9 ± 1.7	41.9 ± 3.3	-	-	-
10		Fe-SMCC № 2	75.3 ± 1.6	31.4 ± 1.3	41.7 ± 2.6	-	-	-
11		Fe-SMCC № 3	55.5 ± 1.2	41.9 ± 0.8	55.5 ± 1.9	-	-	-
12		Fe-SMCC № 4	74.2 ± 1.6	35.5 ± 1.4	47.8 ± 2.9	-	-	-
13		Fe-SMCC № 5	76.0 ± 1.9	30.5 ± 0.9	40.1 ± 2.2	-	-	-
14		Fe-SMCC № 6	77.1 ± 1.8	31.5 ± 1.1	40.9 ± 2.4	-	-	-

## Data Availability

Data are contained within the article.
